# Compound Ciwujia Granules Attenuate Stress‐Induced Anxiety‐ and Depression‐Like Behaviors via Inhibition of TNF‐α‐Associated Neuroinflammation in the dmPFC

**DOI:** 10.1002/cns.71178

**Published:** 2026-09-30

**Authors:** Fei Tian, Yi Li, Mengyu Dai, Yihan Guo, Bingbing Liao, Yingdan Zhang, Xin Gong, Lanying Liu, Dongdong Shi, Zhen Wang

**Affiliations:** ^1^ Shanghai University of Traditional Chinese Medicine Shanghai China; ^2^ Shanghai Mental Health Center Shanghai Jiao Tong University School of Medicine Shanghai China; ^3^ Shanghai Key Laboratory of Psychotic Disorders, Shanghai Mental Health Center Shanghai Jiao Tong University School of Medicine Shanghai China; ^4^ Shanghai Institute of Traditional Chinese Medicine for Mental Health Shanghai Mental Health Center Shanghai China

**Keywords:** Compound Ciwujia Granules, depression, dmPFC, neuroinflammation, TNF signaling pathway

## Abstract

**Aims:**

Compound Ciwujia Granules (CWJ) have shown antidepressant‐like effects, but the neural and molecular mechanisms underlying these effects remain unclear. This study aimed to investigate the mechanisms by which CWJ ameliorates anxiety‐ and depression‐like behaviors.

**Methods:**

A 6‐week chronic unpredictable mild stress (CUMS) model was established in rats, and behavioral tests were performed to assess the antidepressant‐like effects of CWJ. Resting‐state functional magnetic resonance imaging (rs‐fMRI), in vivo fiber photometry, and chemogenetic manipulation were used to identify the brain region and neuronal population involved in the effects of CWJ. Transcriptomic analysis combined with network pharmacology was used to predict candidate molecular pathways, which were further validated by quantitative real‐time polymerase chain reaction, immunofluorescence, western blotting, and pharmacological interventions.

**Results:**

CWJ increased the open‐arm ratio in the EPM, reduced FST immobility, and lowered serum corticosterone (CORT) levels in CUMS rats. Convergent rs‐fMRI, c‐Fos immunostaining, and fiber photometry identified that the dorsomedial prefrontal cortex (dmPFC) was a key brain region involved in the antidepressant‐like effects of CWJ. CWJ increased glutamatergic neuronal activity and reduced neuroinflammatory responses associated with TNF‐related inflammatory signaling in the dmPFC. Moreover, pharmacological activation of TNF‐related inflammatory signaling partially attenuated the behavioral benefits of CWJ, supporting the involvement of  TNF‐related inflammatory signaling in mediating its antidepressant effects.

**Conclusions:**

These findings suggest that CWJ attenuated CUMS‐induced anxiety‐ and depression‐like behaviors by restoring dmPFC glutamatergic neuronal activity and suppressing TNF‐related neuroinflammation in the dmPFC.

## Graphical abstract

By restoring glutamatergic neuronal activity, suppressing TNF‐related inflammatory signaling pathway, and reducing microglial overactivation in the dmPFC, CWJ ameliorated stress‐induced behavioral abnormalities.

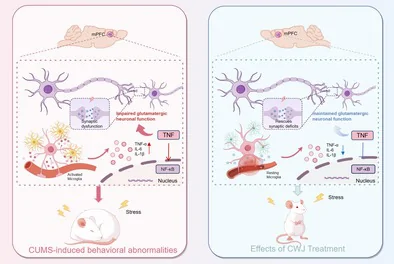

**Abbreviations**
ALFF: amplitude of low‐frequency fluctuation
ASH:
*Acanthopanax senticosus* (Rupr. et Maxim.) Harms
BBB: blood–brain barrier
CORT: corticosterone
CUMS: chronic unpredictable mild stress
CWJ: Compound Ciwujia Granules
ECM: extracellular matrix
ELISA: enzyme linked immunosorbent assay
EPM: elevated plus maze test
FLX: fluoxetine hydrochloride
FST: forced swimming test
HPA: hypothalamic–pituitary–adrenal
Iba1: ionized calcium‐binding adapter molecule 1
IL‐6: interleukin‐6
KEGG: kyoto encyclopedia of genes and genomes
MDD: major depression disorder
mPFC: medial prefrontal cortex
NF‐κB: nuclear translocation of nuclear factor‐κB
OFT: open field test
RS‐fMRI: resting‐state functional magnetic resonance imaging
SC: *Schisandra chinensis*
SPT: sucrose preference test
TCM: Traditional Chinese medicine
TNF: tumor necrosis factor

## 1 Introduction

Major depressive disorder (MDD) is a common psychiatric disorder that contributes substantially to the global burden of disease [1, 2, 3]. Despite the widespread use of first‐line antidepressant therapies, their effectiveness is constrained by delayed therapeutic onset, adverse effects, and insufficient clinical response in many patients [4, 5, 6]. These limitations underscore the need to identify novel and more effective therapeutic approaches for MDD.

An increasing body of evidence implicates immune dysregulation as a key factor in the pathophysiology of depression [3, 7]. Levels of pro‐inflammatory factors, including interleukin (IL)‐1β, IL‐6, and tumor necrosis factor (TNF)‐α, are elevated in patients with depression [8, 9], and these elevations may be attenuated following antidepressant treatment [10, 11, 12]. Furthermore, prolonged inflammation can disrupt neuronal function, thereby affecting central nervous system function and behavior [13]. These findings have stimulated further investigation into the immune‐inflammatory system as a potential target for antidepressant therapy.

Accordingly, the antidepressant effects of traditional Chinese medicine mediated through the modulation of immune and inflammatory pathways have attracted growing interest [14, 15, 16]. Compound Ciwujia Granules (CWJ), consisting of *Acanthopanax senticosus* (Rupr. et Maxim.) Harms (ASH) and *Schisandra chinensis* (SC), have been reported to possess anti‐fatigue, anti‐inflammatory, and neuroprotective properties [17]. Moreover, both clinical and preclinical studies have demonstrated the antidepressant potential of CWJ [18, 19]. However, the neural substrates underlying the antidepressant effects of CWJ remain poorly defined, and its potential immunomodulatory mechanisms in depression have yet to be fully elucidated.

To address these issues, the present study investigated the neural and molecular mechanisms underlying the effects of CWJ. Behavioral tests were used to evaluate the effects of CWJ on chronic unpredictable mild stress (CUMS)‐induced anxiety‐and depressive‐like behaviors. Resting‐state functional magnetic resonance imaging (rs‐fMRI), in vivo fiber photometry, and chemogenetic manipulation were employed to determine the involvement of the dorsomedial prefrontal cortex (dmPFC) in the effects of CWJ. Exploratory transcriptomic and network pharmacology analyses were used to prioritize candidate mechanisms. Molecular and pharmacological experiments subsequently examined the involvement of TNF‐related inflammatory signaling. These findings provide further insight into the neural and inflammatory mechanisms associated with the effects of CWJ on stress‐related behavioral alterations.

## 2 Methods

### 2.1 Drugs and Reagents

CWJ were obtained from Heilongjiang Quanle Pharmaceutical Co. Ltd. (approval no. Z20025148). Fluoxetine (FLX) hydrochloride was also obtained from Heilongjiang Quanle Pharmaceutical Co. Ltd.

### 2.2 Animals

Adult male Sprague–Dawley rats (aged 6–8 weeks) were housed in the experimental holding areas under a 12 h light/dark cycle at 18°C–22°C, with ad libitum access to food and water. All animal procedures were approved by the Institutional Animal Care and Use Committee of Shanghai Jiao Tong University (approval no. A2024334).

### 2.3 Experimental Design

After 1 week of acclimatization, the rats were randomly assigned to four groups: Control (CTRL), CUMS, FLX, and CWJ. Drug interventions began on day 29. Rats in the CTRL and CUMS groups received daily intragastric administration of 0.9% saline at 0.1 mL/100 g body weight. Rats in the CWJ and FLX groups were administered 0.5 g/kg CWJ or 10 mg/kg FLX, respectively. Behavioral tests were conducted from day 43 to day 46, and then blood and brain tissue samples were collected for analysis.

### 2.4 Established CUMS‐Induced Depression Model

Rats were exposed to seven different mild, unpredictable stressors over six consecutive weeks, one stressor per day, including 12 h of water deprivation, 2 h of restraint stress, 12 h of wet bedding, 12 h of cage tilt (45°), 1 min of tail clamping, 12 h of continuous light exposure in a dark cycle, and 5 min of swimming in cold water.

### 2.5 Behavioral Testing Procedures

All behavioral tests were conducted between 8:00 a.m. and 4:00 p.m. Data were recorded and analyzed using Ethovision tracking software (Noldus, Netherlands). Rats were removed from their cages and gently handled for at least 1 min daily for 3 days before testing. The testing order of individual rats from all experimental groups was randomized.

#### 2.5.1 Sucrose Preference Test (SPT)

Two bottles of water were initially placed in the cages for 24 h of acclimatization. Rats were then given two bottles containing a 1% (w/v) sucrose solution for another 24 h. Subsequently, rats were individually housed and deprived of food and water for 6 h. During the test, one bottle with water and one with a 1% sucrose solution were placed in each cage for 3 h, with bottle positions switched once. Consumption volumes were recorded and used for calculating the sucrose preference ratio.

#### 2.5.2 Open Field Test (OFT)

The open field apparatus was a 60 × 60 × 60 cm blue square arena with a central zone of 30 × 30 cm. Rats were placed in the central area in this behavioral test. During the 10‐min test, the total distance moved and the time spent in the central area were recorded.

#### 2.5.3 Elevated Plus Maze Test (EPM)

The device was 60 cm off the ground and contained two open arms (50 × 10 cm) and two closed arms (50 × 10 cm) with a square platform (10 × 10 cm) in the middle. Each rat was placed on the central platform facing an open arm and allowed to explore freely for 5 min. The time spent in each arm and the number of arm entries were recorded.

#### 2.5.4 Forced Swimming Test (FST)

Each rat was placed in a 30 cm × 50 cm transparent cylinder filled with fresh water to a depth of 30 cm. The duration of immobility for each rat was measured during the 5 min of forced swimming.

### 2.6 Resting‐State Functional Magnetic Resonance Imaging and Data Acquisition in Rats

Brain scans were performed in rats from the CTRL, CUMS, and CWJ groups using a Bruker BioSpec 11.7T small‐animal MRI system (Bruker BioSpin, Ettlingen, Germany). Prior to scanning, rats were anesthetized with 5% isoflurane for induction and maintained under 1% isoflurane throughout image acquisition. To minimize anesthesia‐related variability, all animals were scanned using an identical anesthetic protocol. Respiratory activity was continuously monitored throughout image acquisition, and body temperature was maintained between 36°C and 38°C using a circulating warm‐water heating pad. Structural imaging employed a rapid acquisition with relaxation enhancement (RARE) sequence with axial scanning of 42 slices, matrix size of 256 × 256, echo time (TE) of 25 ms, repetition time (TR) of 3500 ms, interslice gap of 0.5 mm, and field of view (FOV) of 25 × 25 mm^2^.

Rs‐fMRI data were preprocessed in MATLAB R2022a (MathWorks, Natick, MA) using SPM12 (http://www.fil.ion.ucl.ac.uk/spm/) and Advanced Normalization Tools (https://github.com/ANTsX/ANTs). Preprocessing included slice‐timing correction, rigid‐body realignment, manual brain extraction, spatial normalization to the SIGMA rat brain template using linear registration and ANTs symmetric normalization, spatial smoothing, linear detrending, regression of white‐matter and cerebrospinal‐fluid signals, and band‐pass filtering at 0.01–0.08 Hz. Results were corrected for multiple comparisons using family‐wise error rate (FWER) correction at *p* < 0.05. An additional spatial‐extent criterion of more than 20 contiguous voxels was applied.

### 2.7 Immunohistochemistry and Imaging

Brain tissues were collected after the completion of all behavioral tests. No additional experimental stimulation was administered during this interval. Samples from all experimental groups were processed, immunostained, imaged, and quantified in the same batch under identical experimental and image acquisition conditions. The brain tissue was cut into 30 μm thick slices using a cryostat (CM1950, Leica) and mounted on slides. Sections were blocked with QuickBlock blocking buffer (P0260, Beyotime Biotechnology) at room temperature (RT) for 10 min, and then incubated overnight at 4°C with rabbit anti‐c‐Fos antibody (1:2000; ab190289, Abcam), rabbit anti‐ionized calcium‐binding adapter molecule 1 (Iba1) antibody (1:1000; 019‐19741, FUJIFILM Wako), or mouse anti‐CaMKIIα antibody (1:50; sc‐13141, Santa Cruz Biotechnology Inc.). The following day, sections were incubated with secondary antibodies as follows: anti‐mouse IgG (H + L), Alexa Fluor 488 (1:1000; A‐11001), anti‐rabbit IgG (H + L), Alexa Fluor 555 (1:1000; A‐31572), and anti‐rabbit IgG (H + L), Alexa Fluor 647 (1:1000; A‐21245, all Thermo Fisher Scientific) at room temperature for 1.5 h. Images were acquired using an Olympus VS200 slide scanner and a FLUOVIEW FV1200 confocal microscope (Olympus). Image analysis was performed using Fiji software (NIH, Bethesda MD, USA).

### 2.8 Quantitative Real‐Time Polymerase Chain Reaction (qRT‐PCR)

Total RNA was extracted using QIAzol lysis reagent and purified with the RNeasy Mini Kit (QIAGEN) as described previously [20]. Reverse transcription was performed using a cDNA kit (Life Technologies), and then TB Green Premix Ex Taq (Takara Bio) was used to perform qRT‐PCR. Fluorescence signals were detected and analyzed using a LightCycler 480 system (Roche Diagnostics).

### 2.9 RNA Sequencing Analysis

RNA sequencing was performed using dmPFC samples from 4 rats per group. Each sequencing library was generated from one individual rat, and no samples were pooled. After RNA extraction, read count normalization and gene expression estimation were performed using HTSeq. Differential expression analysis was conducted with limma‐voom pipeline. Since only four of the genes survived FDR correction, genes meeting the criteria of *p* < 0.05, and | log_2_(Fold Change) | > 0.585 were defined as differentially expressed genes (DEGs) for exploratory screening experiment and downstream pathway analysis. Enrichment analyses were performed using R version 4.2.0. 

### 2.10 Enzyme‐Linked Immunosorbent Assays (ELISA)

Blood samples were allowed to coagulate for 30 min at room temperature after collection. The coagulated blood was then centrifuged at 3000× *g* for 20 min at 4°C. The serum supernatant was carefully aspirated and stored at −80°C until further analysis. The ELISA was performed according to the manufacturer's instructions (EK8100, Multisciences Biotech Co. Ltd., China) to quantify serum corticosterone (CORT) concentrations. Optical density values were measured within 30 min at 450 nm.

### 2.11 Western Blotting

To determine protein expression, dmPFC tissue was homogenized in RIPA buffer containing 1% PMSF (P0013B, Beyotime). After standing at 4°C for 30 min, the supernatant was centrifuged and the protein concentration was determined using a BCA kit (P0012, Beyotime). Proteins were separated on 4%–12% SDS‐PAGE gels and transferred to PVDF membranes (0.45 μm, Beyotime). The membranes were blocked with Quick Blocking buffer (TBSTw, Beyotime) at RT for 15 min. After overnight incubation with rabbit monoclonal anti‐nuclear factor kappa B (NF‐κB; 1:1000; 8242, Cell Signaling Technology), rabbit monoclonal anti‐phospho‐NF‐κB (1:1000; 3033, Cell Signaling Technology), rabbit polyclonal anti‐TNF‐α (1:1000; 17590‐1‐AP, Proteintech), or anti‐*β*‐actin (1:5000; 3450S, Cell Signaling Technology) antibodies at 4°C, then incubated membranes with HRP‐linked anti‐rabbit IgG (1:5000; 7074, Cell Signaling Technology) for 1 h at room temperature. Proteins were detected using an ECL kit (P0018FS, Beyotime) and quantified using Fiji software.

### 2.12 Transmission Electron Microscope (TEM)

After perfusion, the dmPFC was cut into 1 mm^3^ tissue blocks, fixed in 2.5% glutaraldehyde for 6 h, and stored at 4°C. After rinsing with 0.1 M PB and ultrapure water, the samples were dehydrated through a graded ethanol series and embedded in resin. An ultramicrotome (Ultracut UCT, Leica) was used to cut 0.4 μm sections and place them on a copper grid. During imaging, the samples were placed on a special copper mesh and rapidly frozen in liquid nitrogen. Images were collected using a transmission electron microscope (Tecnai G2 spirit Biotwin, Thermofisher). All eligible fields were acquired using identical settings, and all clearly identifiable synapses with a discernible postsynaptic density (PSD) were analyzed. Synaptic measurements were averaged within each rat, and the rat was treated as the experimental unit.

### 2.13 Stereotaxic Surgery and Virus Injection

These experiments were performed as described before [21]. Rats were anesthetized with isoflurane (RWD, China) and secured in a stereotaxic apparatus (RWD, China). After the skull was exposed, 0.5 μL of virus‐containing solution was injected at 0.067 μL/min using a 33‐gauge needle (Hamilton). The injection coordinates followed the rat brain atlas for the dmPFC (AP: +2.70 mm; ML: ±0.40 mm; DV: −3.20 mm).

For fiber photometry experiments, recombinant adeno‐associated virus (rAAV)‐CaMKIIα‐GCaMP6s‐WPRE‐hGH polyA virus was infused into the dmPFC. For chemogenetic behavioral experiments, AAV‐CaMKIIα‐hM3D(Gq)‐mCherry‐WPRE or AAV‐CaMKIIα‐hM4D(Gi)‐mCherry‐WPRE‐hGH polyA was bilaterally injected. AAV‐CaMKIIα‐mCherry injections matched the experimental group. Rats recovered for 3 weeks before behavioral tests. Animals with inaccurate injection sites were excluded. All adeno‐associated viruses (AAVs) were purchased from BrainVTA Technology Co. Ltd. (China). To activate or inhibit dmPFC neurons, clozapine‐*N*‐oxide (CNO; S6887, Selleck) was dissolved in saline and administered at a dose of 1 mg/kg intraperitoneally 30 min before behavioral tests.

### 2.14 Optical‐Fiber‐Based Ca^2+^ Recording

The Ca^2+^ signals were recorded using an Inper fiber‐optic photometer (Inper, Hangzhou, China). An optical fiber (NA 0.37, Inper) with a diameter of 200 μm was inserted into the right dmPFC. Inper Studio was used to simultaneously record neuronal Ca^2+^ signals and behavioral videos, and Inper Data Process was used for data analysis.

### 2.15 In Vivo Drug Administration

Infliximab (INF, HY‐P9970, MCE) was dissolved in saline at 100 ng/μL. A 0.5 μL dose of vehicle or INF was delivered bilaterally into the dmPFC via an implanted guide cannula (RWD) 2 h before behavioral tests.

Resiquimod (R848, S8133, Selleck) was dissolved in 1% DMSO, then diluted in saline to a final concentration of 100 ng/μL. A 0.5 μL dose of vehicle or R848 was delivered bilaterally into the dmPFC via implanted guide cannula (RWD) 2 h before behavioral tests.

### 2.16 UPLC‐Q‐Exactive Orbitrap Mass Spectrometry

Chromatographic separation used an ACQUITY UPLC HSS T3 column (2.1 × 100 mm, 1.8 μm; Waters, Milford, MA, USA) with a gradient elution of 0.1% formic acid in deionized water (A) and 0.1% formic acid in acetonitrile (B): 0–0.5 min, 98% A; 0.5–2 min, 95% A; 2–3 min, 85% A; 3–13 min, 50% A; 13–25 min, 5% A; 25–30 min, 5% A; 30–30.5 min, 95% A; 30.5–35 min, 95% A. The column temperature was 35°C, the flow rate was 0.3 mL/min, and the injection volume was 10 μL.

Mass spectrometry employed an electrospray ionization (ESI) source and a Fourier transform (FT) mass analyzer in data‐dependent acquisition (DDA) mode. Key parameters were full scan (MS^1^) resolution of 60,000, MS^2^ resolution of 15,000, and an *m*/*z* range of 50–1500.

### 2.17 Network Pharmacology Analysis of CWJ Components

The CWJ component target database was established by retrieving data on active compounds and their predicted targets from the TCMSP (https://www.tcmsp‐e.com/) and SwissTargetPrediction (https://www.swisstargetprediction.ch/) databases. The depression disease target database was compiled by screening for depression‐related targets using the OMIM (https://omim.org/), GeneCards (http://www.genecards.org/), and DisGeNET (https://disgenet.com/) databases. Intersection analysis between CWJ components and depression targets was performed using Venny 2.1 (https://bioinfogp.cnb.csic.es/tools/venny/), followed by visualization of the resulting network. The protein–protein interaction (PPI) network was constructed using the STRING database (https://string‐db.org/) and visualized using Cytoscape 3.7.2.

### 2.18 Statistical Analysis

Data analysis and visualization were performed using GraphPad Prism 9.5 (GraphPad Software, San Diego, USA). Results are expressed as the mean ± standard error of the mean. Statistical significance between two groups for normally distributed data was assessed using Student's two‐tailed *t*‐test. One‐way ANOVA accompanied by Tukey's post hoc test was employed for comparative analysis among multiple groups. Nonparametric tests were utilized when the data had a non‐normal distribution. A *p* < 0.05 indicates statistical significance, while “ns” indicates no statistical significance.

## 3 Results

### 3.1 CWJ Attenuates CUMS‐Induced Anxiety‐ and Depression‐Like Behaviors

To evaluate the behavioral and neuroendocrine effects of CWJ, rats were exposed to CUMS and subsequently treated with CWJ, with FLX serving as a positive control (Figure 1a). CUMS did not affect the total distance traveled by the animals (Figure 1d,e), indicating that CUMS did not alter locomotor function. In the EPM, CUMS significantly reduced the open arm ratio; whereas both CWJ and FLX reversed this change (Figure 1f). In the SPT, one‐way ANOVA showed a significant overall group effect on the sucrose preference ratio. However, post hoc comparisons showed that the decrease in sucrose preference ratio in the CUMS group compared with the CTRL groupdid not reach statistical significance. Similarly, CWJ produced a numerical increase in the sucrose preference ratio compared with the CUMS group, but this difference was not statistically significant (Figure 1g). In the FST, CWJ and FLX significantly reduced the immobility duration compared to the CUMS rats (Figure 1h).

**FIGURE 1:**
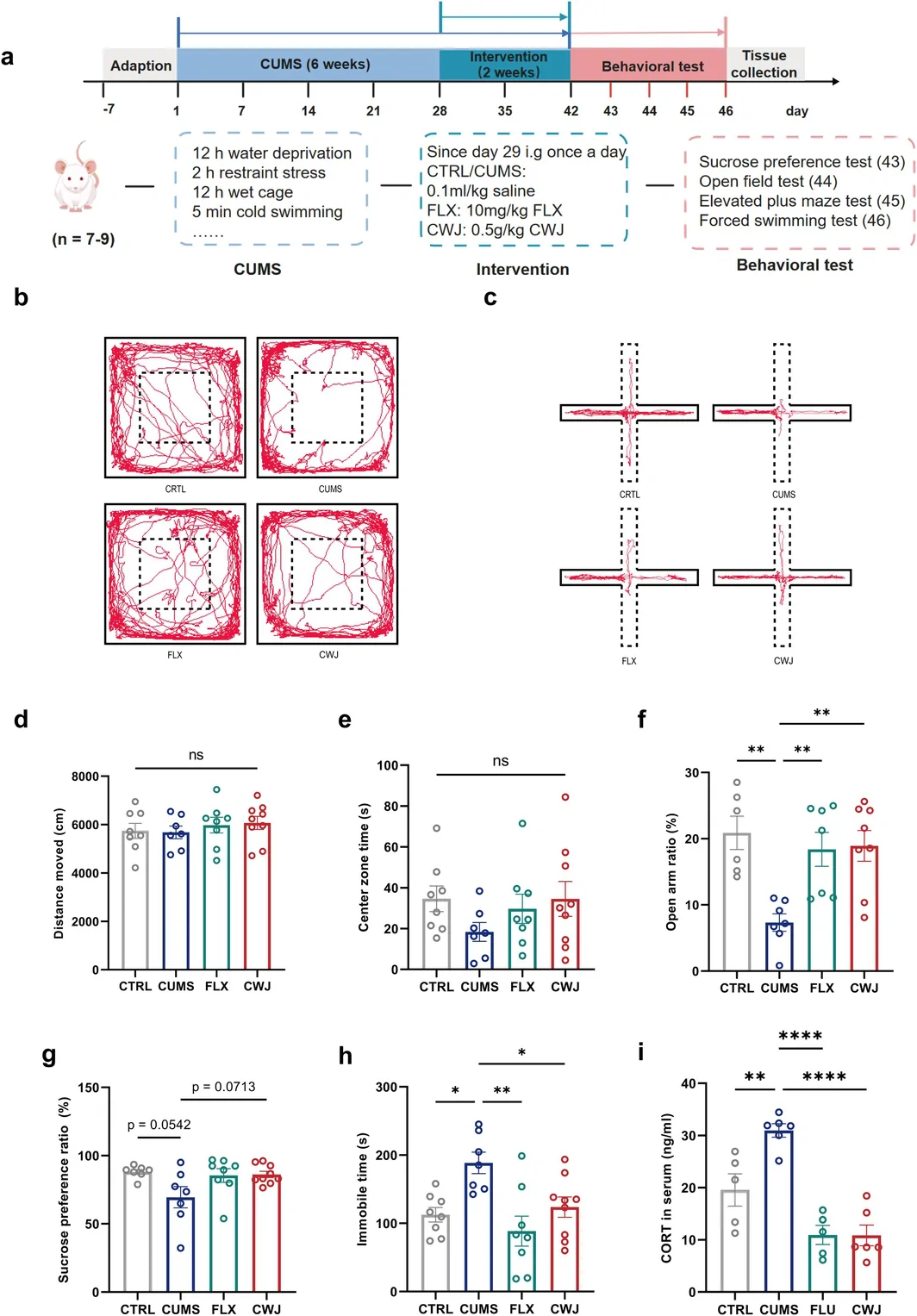
Effects of CWJ on CUMS‐induced behavioral abnormalities and serum corticosterone levels. (a) Schematic illustration of the experimental design. Behavioral tests were conducted following a fixed and standardized test sequence: Sucrose preference test (day 43), open field test (day 44), elevated plus maze (day 45), forced swimming test (day 46). (b) Representative movement trajectories of rats in each group in the OFT. (c) Representative movement trajectories of rats in each group in the EPM. (d) Time spent in the center zone in the OFT. [One‐way ANOVA, *F*(3, 28) = 1.090, *p* = 0.3695; *n* = 7–9 in each group]. (e) Total distance traveled in the OFT. [One‐way ANOVA, *F*(3, 28) = 0.4048, *p* = 0.7507; *n* = 7–9 in each group]. (f) Open arm ratio in the EPM. [One‐way ANOVA, *F*(3, 25) = 5.720, *p* = 0.0040; *n* = 6–9 in each group]. (g) Sucrose preference ratio in the SPT. [One‐way ANOVA, *F*(3, 27) = 3.199, *p* = 0.0392; Tukey's multiple comparisons test, CTRL vs. CUMS, *p* = 0.0542, CUMS vs. CWJ, *p* = 0.0713, *n* = 7–9 in each group]. (h) Immobility time in the FST. [One‐way ANOVA, *F*(3, 28) = 6.259, *p* = 0.0022; *n* = 7–9 in each group]. (i) Serum corticosterone (CORT) levels. [One‐way ANOVA, *F*(3, 18) = 22.13, *p* < 0.0001; *n* = 5–6 in each group].

Dysfunction of the hypothalamic–pituitary–adrenal (HPA) axis is commonly observed in patients with depression [22, 23, 24]. Therefore, we measured the serum CORT concentration. CUMS significantly increased the CORT levels, whereas both CWJ and FLX decreased the CORT concentrations (Figure 1i). Collectively, CWJ attenuated CUMS‐associated alterations in the EPM, FST, and serum CORT levels. However, its effect on the sucrose preference ratio was not statistically significant. These findings indicate that CWJ ameliorated stress‐related behavioral and neuroendocrine abnormalities rather than reversing all dimensions of the CUMS phenotype.

### 3.2 Identification of the dmPFC as a Key Brain Region Mediating the Effects of CWJ

Although previous studies have demonstrated that components of CWJ exert therapeutic effects in the brain [25], to our knowledge, it is not clear whether CWJ simultaneously acts on many brain regions or only a few specific sites to improve depressive‐like behavior. To characterize the impact of CWJ on regional spontaneous brain activity in a CUMS‐induced depression model, rs‐fMRI was performed to detect spontaneous hemodynamic responses throughout the brain (Figure 2a) [26]. The intensity of spontaneous neural activity was assessed using zALFF. Compared with the CTRL group, the CUMS group exhibited significantly decreased zALFF in the secondary auditory cortex and primary motor cortex (Figure 2b, Table 1). In contrast, the CWJ‐treated group showed increased zALFF in the parietal cortex, prelimbic cortex (PrL), and orbitofrontal region compared to the CUMS group (Figure 2c, Table 1). Based on the above results and the findings of previous studies [27], we hypothesized that dmPFC may be a key brain region involved in the effects of CWJ on CUMS‐induced behavioral abnormalities. 

**FIGURE 2:**
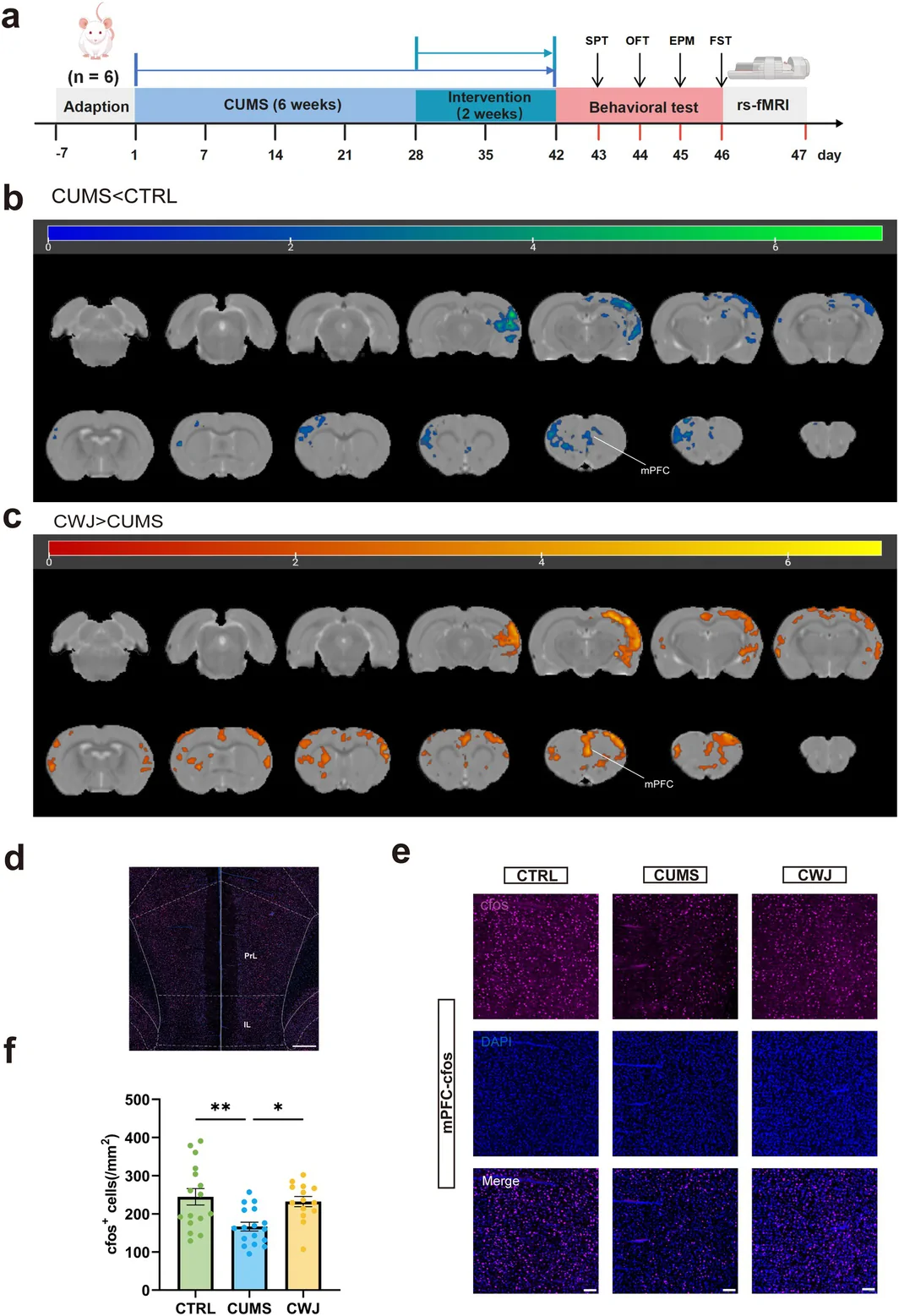
Effects of CWJ on brain functional activity in CUMS rats. (a) Schematic illustration of the experimental design. (b) ALFF maps showing reduced brain activity in the CUMS group compared with the CTRL group (*n* = 5–6 rats in each group). (c) ALFF maps showing partial restoration of the altered activity pattern after CWJ treatment compared with the CUMS group (*n* = 5–6 rats in each group). (d, e) Representative immunofluorescence images of c‐Fos expression in the dmPFC. Scale bars: 500 and 100 μm. (f) Quantification of c‐Fos^+^ cells in the PrL [one‐way ANOVA, *F*(2, 44) = 7.043, *p* = 0.0022; *n* = 6 in each group].

**TABLE 1:** Regions showing ALFF differences between CTRL and CUMS, and between CWJ and CUMS.

	Voxel size	Peak mini coordinate (mm)			*t*‐value
		*X*	*Y*	*Z*	
CTRL > CUMS					
Secondary_Auditory_Cortex_Dorsal_Part_L	1104	−6.7	3.62	−5.305	8.465
Primary_Motor_Cortex_R	896	2.6	5.42	4.295	5.2808
CWJ > CUMS					
Parietal_cortex_postero_rostral_L	384	−6.1	4.82	−4.105	7.6793
PreLimbic_system_L	106	−0.4	3.02	3.095	6.9902
Orbitofrontal_region_L	126	−4	5.12	3.995	7.54

Subsequently, we examined c‐Fos expression in the dmPFC (Figure 2d–f). The results showed that CUMS exposure significantly decreased c‐Fos expression in the dmPFC, whereas CWJ treatment partially reversed this reduction. Together with the rs‐fMRI findings, these results provided complementary evidence that CWJ was associated with the partial restoration of stress‐related functional alterations in the dmPFC.

### 3.3 CWJ Improves Stress‐Related Behaviors Partly Through Enhanced dmPFC Glutamatergic Neuronal Activity

Previous studies have implicated the mPFC in depression in both patients and rodent models [28], and our rs‐fMRI results showed altered dmPFC activity after CUMS exposure. We therefore examined whether dmPFC glutamatergic neurons contribute to the behavioral effects of CWJ. To monitor neuronal activity, AAV‐CaMKIIα‐GCaMP6s was injected into the dmPFC, and calcium signals were recorded during the FST (Figure 3a–c). During struggling bouts, CUMS‐exposed rats showed significantly lower Ca^2+^ signals than CTRL rats, whereas CWJ treatment increased these signals relative to CUMS (Figure 3d–g).

**FIGURE 3:**
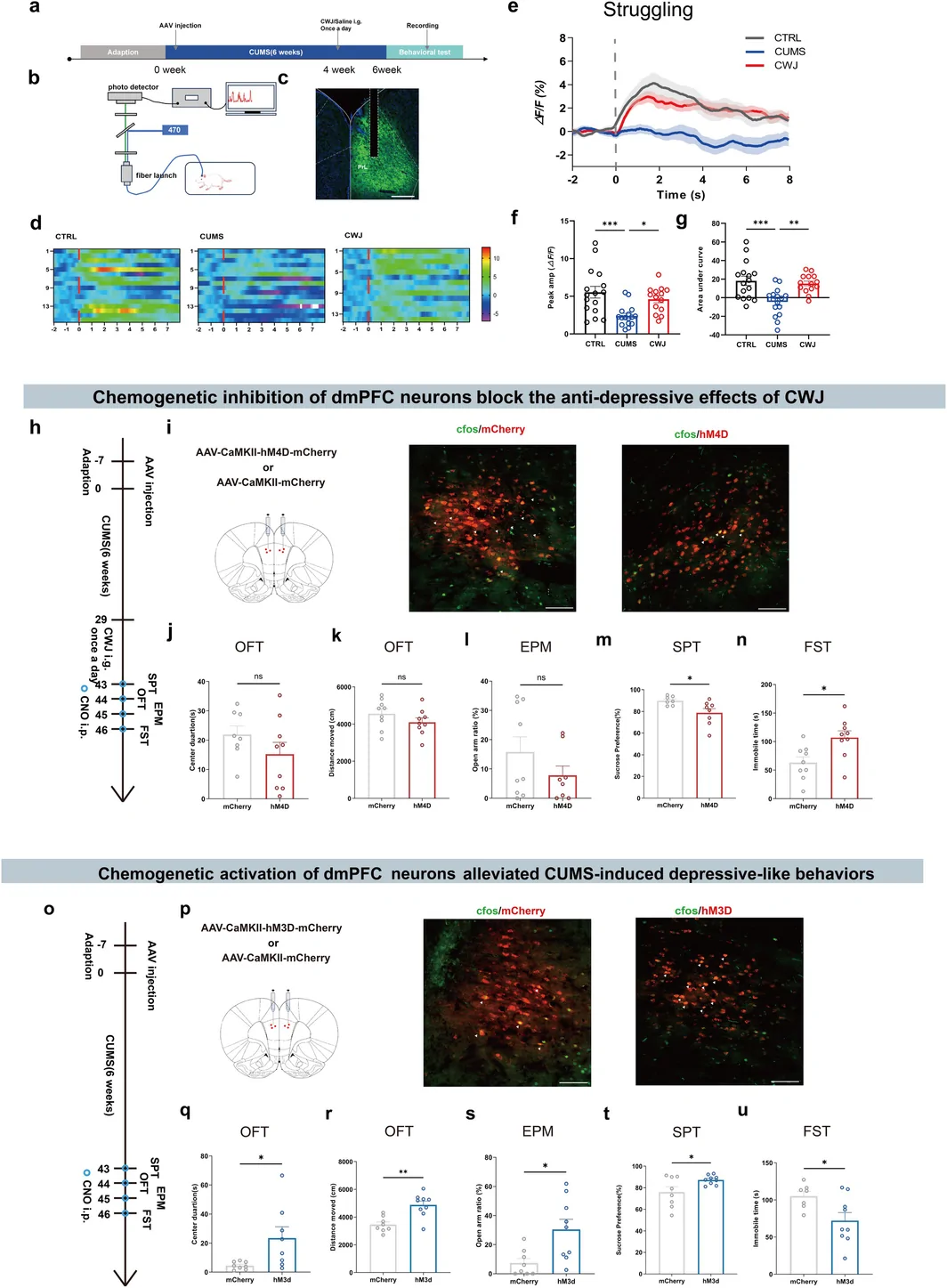
CWJ mitigated depressive‐like behaviors by enhancing glutamatergic neuronal activity in the dmPFC. (a) Schematic illustration of the experimental design for in vivo fiber photometry recording. (b) Schematic diagram of the fiber photometry setup. (c) Representative image of optical fiber implantation in the mPFC targeting glutamatergic neurons expressing GCaMP6. Scale bar: 500 μm. (d) Heatmaps of Δ*F*/*F* illustrating Ca^2+^ signals during FST struggling events; time 0 indicates event onset (*n* = 5–6 in each group). (e) Δ*F*/*F* changes in Ca^2+^ signals during struggling events. Solid lines and shaded areas represent the mean ± standard error. (f) Quantification of the peak amplitude of neuronal Ca^2+^ signals [one‐way ANOVA, *F*(2, 43) = 8.526, *p* = 0.0008; *n* = 5–6 in each group]. (g) Quantification of the AUC(Δ*F*/*F*%) from struggling behavior [one‐way ANOVA, *F*(2, 43) = 9.938, *p* = 0.0003; *n* = 5–6 in each group]. (h) Schematic of the experimental timeline and behavioral paradigms. (i) Left: Schematic illustration of the virus injection into the bilateral dmPFC. Right: Representative image of c‐Fos^+^ and mcherry cells. Scale bar: 100 μm. (j) The time spent in the central zone during the OFT is not significantly different (*t*‐test, *p* = 0.2030, *n* = 9). (k) No significant difference in total distance traveled between the hM4D and mCherry groups (*t*‐test, *p* = 0.2120, *n* = 9). (l) No significant difference in the open arm ratios in the EPM (Mann–Whitney test, *U* = 25, *p* = 0.3199). (m) Sucrose preference ratio in the SPT (*t*‐test, *p* = 0.0260, *n* = 9). (n) Immobility time in the FST (*t*‐test, *p* = 0.5039, *n* = 9). (o) Schematic of the experimental timeline and behavioral paradigms. (p) Left: Schematic illustration of the virus injection into the bilateral dmPFC. Right: Representative image of c‐Fos^+^ and mcherry cells. Scale bar: 100 μm. (q) Time spent in the central zone during the OFT (*t*‐test, *p* = 0.0226, *n* = 8–9). (r) The hM3D group traveled a significantly greater distance than the mCherry control group (*t*‐test, *p* = 0.0011, *n* = 8–9). (s) The hM3D group shows a significantly higher open arm ratio than the mCherry control group (*t*‐test, *p* = 0.0125, *n* = 8–9). (t) The hM3D group shows a significantly higher sucrose preference ratio than the mCherry control group (*t*‐test, *p* = 0.0376, *n* = 8–9). (u) Immobility time in the FST (*t*‐test, *p* = 0.0272, *n* = 8–9).

We next used bidirectional chemogenetic manipulation to examine the functional contribution of these neurons. To determine whether dmPFC glutamatergic neurons contribute to the antidepressant‐like effects of CWJ, we inhibited these neurons using hM4D(Gi) after CWJ treatment. Chemogenetic inhibition did not significantly affect locomotor function or open arm ratios, but it attenuated the effects of CWJ on depressive‐like behavior, including the sucrose preference ratio in the SPT and the immobility time in the FST (Figure 3h–n). We next tested whether the activation of dmPFC glutamatergic neurons was sufficient to ameliorate CUMS‐induced behavioral abnormalities. Activation of these neurons with hM3D(Gq), followed by CNO administration 30 min before behavioral testing, improved several stress‐related behavioral measures compared to those in the mCherry control group (Figure 3o–u). Because the total distance traveled in the OFT differed between groups, we further analyzed the center‐zone duration in the OFT using the total distance traveled as a covariate. This ANCOVA analysis showed that the group effect of center‐zone duration was no longer significant after adjustment for total distance (Figure S1). Together, the fiber‐photometry and chemogenetic findings indicate that dmPFC glutamatergic neuronal activity contributes to the behavioral effects of CWJ, particularly those reflected by sucrose preference and FST immobility.

### 3.4 Transcriptomic and Network Pharmacology Analyses Implicate TNF‐Associated Signaling in the Effects of CWJ

To explore transcriptomic alterations in the dmPFC associated with CUMS exposure and CWJ treatment, RNA sequencing was performed using four independent rats per group. Differential expression analysis identified 339 DEGs between the CUMS and CTRL groups and 3417 between the CWJ and CUMS groups (Figure 4a,b). Gene Ontology (GO) enrichment linked the CUMS‐responsive genes to ECM‐related terms and the CWJ‐responsive genes to ECM structural constituents (Figure 4f,h). Because a fundamental characteristic of the ECM is its capacity for dynamic remodeling in response to inflammatory stimuli, tissue injury, or infection [29], these findings suggested the involvement of inflammatory processes. Kyoto Encyclopedia of Genes and Genomes (KEGG) pathway analysis also identified inflammation‐associated pathways in both comparisons (Figure 4g,i). For exploratory target prioritization, the altered genes from the two comparisons were intersected, yielding 123 overlapping genes. Exploratory enrichment analysis highlighted glutamate receptor activity and pathways related to neuroactive ligand–receptor interactions, calcium, MAPK, and TNF signaling (Figure 4d,e), providing candidate mechanisms for subsequent evaluation.

**FIGURE 4:**
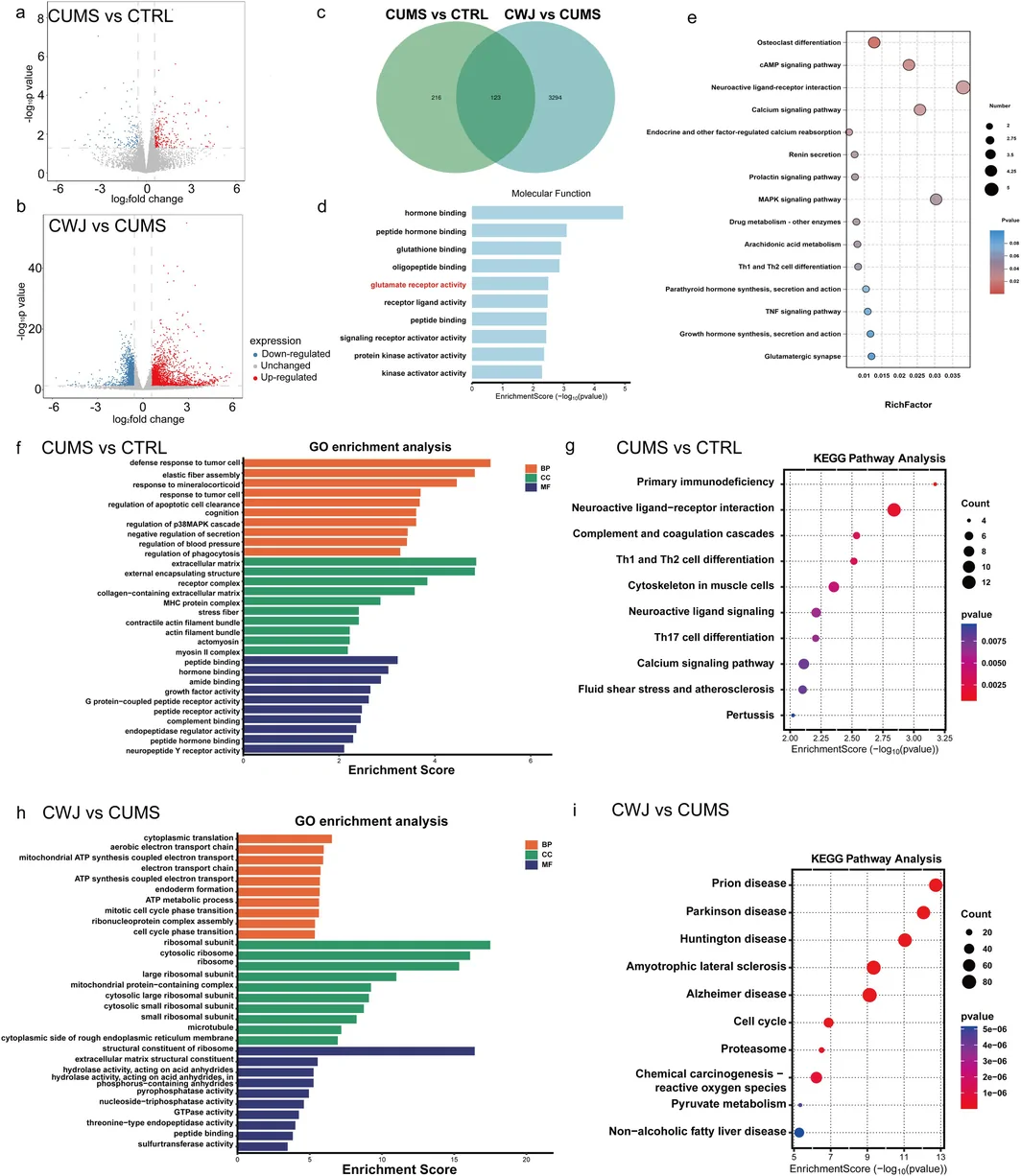
Transcriptomic profiling identifies inflammation‐related pathways associated with the antidepressant‐like effects of CWJ. (a) Volcano plot illustrating differentially expressed genes (DEGs) between CTRL and CUMS groups. (b) Volcano plot illustrating DEGs between CUMS and CWJ groups. (c) Venn diagram of overlapping DEGs among groups. (d) Molecular function analysis of DEGs in the overlapping DEGs. (e) KEGG pathways enriched in the overlapping DEGs shown in the Venn diagram. (f) Gene Ontology (GO) enrichment analysis of DEGs between the CTRL and CUMS groups. (g) KEGG pathways between the CTRL and CUMS groups. (h) GO terms of pathways between CUMS and CWJ groups. (i) KEGG pathways between CUMS and CWJ groups.

HPLC‐Q‐TOF/MS identified 29 putative constituents of CWJ (Table 2 and Figure S2). Network pharmacology analysis based on these constituents identified 409 potential therapeutic targets, with TNF, IL6, INS, IL1B, and SRC emerging as major hub nodes in the PPI network (Figure S3a,b). Enrichment analysis further highlighted inflammatory and synaptic processes, including MAPK, cAMP, calcium, dopaminergic synapse, and TNF signaling pathways (Figure S3c,d). Together, the convergence of the exploratory transcriptomic and network pharmacology findings prioritized TNF‐related inflammatory signaling for subsequent molecular and pharmacological evaluation.

**TABLE 2:** The components of CWJ.

Mode	Identification	RT (min)	*m*/*z*	Reference ion
ESI+	Schisandrin	14.618	415.2099	[M + H−H_2_O]+
ESI+	Schisandrin B	20.659	401.1946	[M + H]+
ESI+	Adenosine	1.661	268.1034	[M + H]+
ESI+	Isofraxidin	6.472	223.0595	[M + H]+
ESI+	Schizandrin A	19.922	417.2260	[M + H]+
ESI+	Cinnamaldehyde	6.072	133.0642	[M + H]+
ESI+	L‐Valine	0.890	118.0857	[M + H]+
ESI+	Isoferulic acid	5.685	177.0542	[M + H−H_2_O]+
ESI+	Schisanhenol	17.625	403.2102	[M + H]+
ESI+	Gomisin J	15.271	389.1946	[M + H]+
ESI+	3,4‐Dihydroxybenzaldehyde	3.473	171.0647	[M + H + MeOH]+
ESI+	7‐Methoxy‐4‐methylcoumarin	7.473	191.0699	[M + H]+
ESI+	Vanillic acid	4.229	169.0492	[M + H]+
ESI+	Schisandrin C	21.146	385.1634	[M + H]+
ESI+	Fraxetin	6.425	209.0441	[M + H]+
ESI+	Sinapic acid	6.000	225.0752	[M + H]+
ESI+	Gomisin D	15.172	569.1765	[M + K]+
ESI−	Citric acid	1.308	191.0195	[M − H]−
ESI−	Cryptochlorogenic acid	6.139	353.0867	[M − H]−
ESI−	Eleutheroside B	6.069	417.1387	[M + FA − H]−
ESI−	Protocatechuic acid	3.841	153.0193	[M − H]−
ESI−	Sucrose	0.904	377.0847	[M + Cl]−
ESI−	Caffeic acid	6.231	179.0348	[M − H]−
ESI−	Azelaic acid	9.137	187.0972	[M − H]−
ESI−	Salicylic acid	9.226	137.0245	[M − H]−
ESI−	4‐Methoxysalicylic acid	4.295	167.0348	[M − H]−
ESI−	Isoferulic acid	6.549	387.1073	[2M − H]−
ESI−	Isochlorogenic acid B	8.443	515.1177	[M − H]−

### 3.5 CWJ Mitigates Neuroinflammation in the dmPFC Through the TNF Signaling Pathway

To investigate the anti‐inflammatory mechanisms of CWJ in the dmPFC, we measured the mRNA expression of inflammatory‐related genes by RT‐qPCR (Table S1). CWJ administration significantly decreased *Ifng*, *Il6*, and *Tnf* expression while increasing *Il10* expression in the dmPFC (Figure S4). Consistent with these transcriptional changes, CUMS increased TNF‐α, NF‐κB, and phosphorylated NF‐κB protein levels, whereas CWJ significantly attenuated these increases (Figure 5a,b). These molecular findings support an inhibitory effect of CWJ on TNF/NF‐κB‐related inflammatory signaling.

**FIGURE 5:**
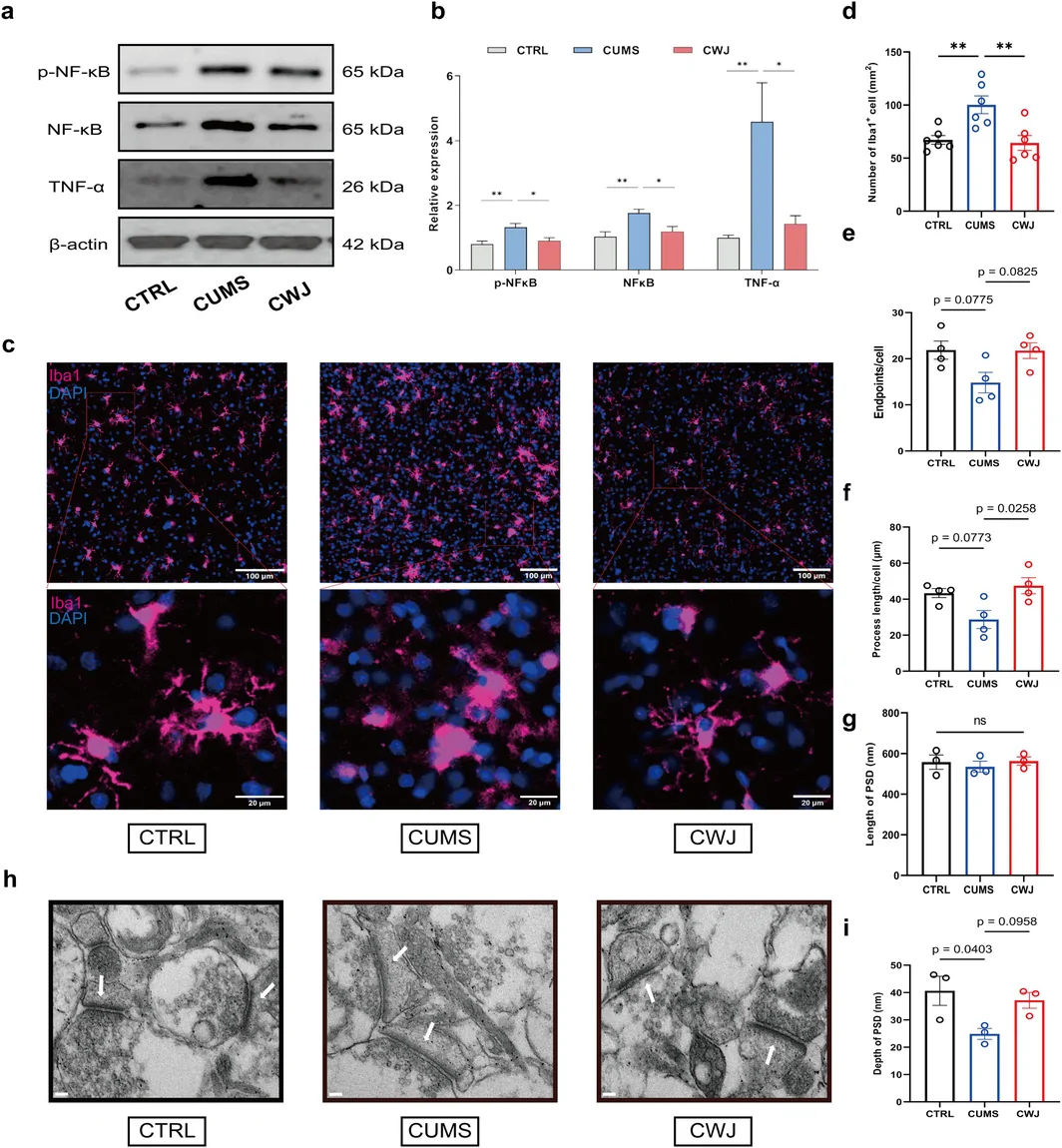
Effects of CWJ on TNF‐related signaling, microglial changes, and synaptic ultrastructure in the dmPFC. (a) Western blot analysis of TNF and NF‐𝜅B signaling pathways in the mPFC. (b) Quantification of protein levels in the dmPFC (*n* = 3 in each group). (c) Representative immunofluorescence images of Iba1‐positive microglia (magenta) and DAPI‐stained nuclei (blue) in the dmPFC. Scale bars: 100 and 20 μm. (d) Quantification of Iba1‐positive cells. [One‐way ANOVA, *F*(2, 15) = 8.818, *p* = 0.0029; *n* = 6 in each group]. (e) Analysis of microglial endpoints. [One‐way ANOVA, *F*(2, 9) = 4.133, *p* = 0.0533; *n* = 4 in each group]. (f) Analysis of microglial process length. [One‐way ANOVA, *F*(2, 9) = 5.721, *p* = 0.0249; *n* = 4 in each group]. (g) Quantification of postsynaptic density length. [One‐way ANOVA, *F*(2, 6) = 0.2716, *p* = 0.7711; *n* = 3 in each group]. (h) Representative electron micrographs showing synaptic structures and postsynaptic densities in the dmPFC. Scale bar: 0.2 μm. (i) Quantification of postsynaptic density thickness. [One‐way ANOVA, *F*(2, 6) = 5.079, *p* = 0.0512; *n* = 3 in each group].

In the central nervous system, TNF regulates homeostasis by modulating neurogenesis, myelination, and synaptic plasticity [30], but it can also increase neuronal excitotoxicity and promote neuroinflammation. Neuroinflammation is mediated by microglial activation [31]. We used immunofluorescence staining for the microglia‐specific marker ionized calcium‐binding adapter molecule 1 (Iba‐1) and quantified microglial morphological parameters in the dmPFC [32]. CUMS significantly increased the number of Iba1‐positive cells, whereas CWJ significantly attenuated this increase (Figure 5d). The number of microglial process endpoints did not differ significantly between CTRL and CUMS or between CUMS and CWJ (Figure 5e). Microglial process length was numerically reduced by CUMS but was significantly increased by CWJ relative to CUMS (Figure 5f). TEM analysis showed no significant group difference in PSD length (Figure 5g). Although a pairwise comparison suggested reduced PSD depth in CUMS rats relative to CTRL rats, the CUMS‐versus‐CWJ comparison was not significant (Figure 5i). Collectively, CWJ attenuated selected CUMS‐associated microglial changes, whereas its effect on synaptic ultrastructure remained inconclusive.

### 3.6 Pharmacological Modulation of TNF‐Associated Signaling Alters Stress‐Related Behaviors and dmPFC Glutamatergic Neuronal Activation

To examine the involvement of TNF‐associated inflammatory signaling, we applied complementary pharmacological manipulations in the dmPFC. Resiquimod (R848), an agonist associated with TNF induction [33, 34], was administered into the dmPFC 2 h before behavioral testing (Figure 6a). Compared with the CWJ group, R848 administration did not significantly alter center‐zone duration or total distance traveled in the OFT (Figure 6b,c). However, R848 significantly reduced the open arm ratio in the EPM and the sucrose preference ratio in the SPT (Figure 6d,e). R848 also increased dmPFC *Tnf* expression and reduced the number of c‐Fos/CaMKIIα co‐labeled cells (Figure S5a and Figure 6f,g), indicating reduced activity‐related labeling in CaMKIIα‐positive neurons. These findings suggest that R848‐induced inflammatory signaling partially counteracted the behavioral and neuronal effects of CWJ.

**FIGURE 6:**
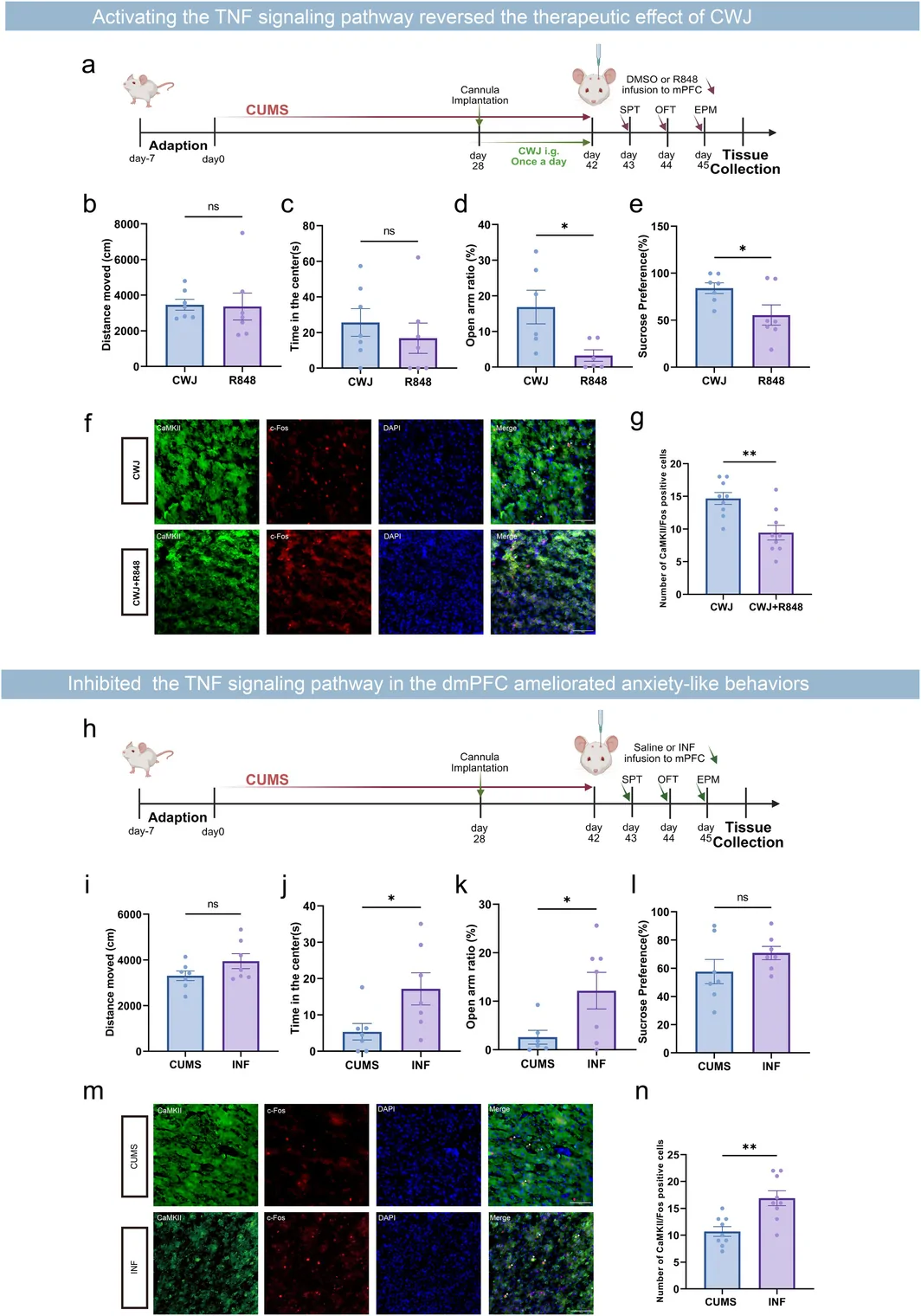
Modulation of TNF‐related signaling influences the behavioral effects of CWJ in CUMS rats. (a) Schematic illustration of the experimental design for activation of TNF‐related signaling. (b) Duration spent in the central zone during the OFT [Mann–Whitney test, *p* = 0.3980; *n* = 7 in each group]. (c) Distance moved in the OFT [Mann–Whitney test, *p* = 0.3176; *n* = 7 in each group]. (d) Open arm ratio in the EPM [Mann–Whitney test, *p* = 0.0260; *n* = 6 in each group]. (e) Sucrose preference ratio in the SPT [*t*‐test, *p* = 0.0367; *n* = 7 in each group]. (f) Representative images showing CaMKII^+^ neurons, c‐Fos^+^ neurons, and co‐localized cells in the PrL of each group. Scale bar: 100 μm. (g) Number of c‐Fos^+^ neurons co‐localized with CaMKII^+^ neurons (*t*‐test, *p* = 0.0024; *n* = 3 slices from 3 rats per group). (h) Schematic illustration of the experimental design for inhibition of TNF‐related signaling. (i) Distance moved in the central zone during the OFT (*t*‐test, *p* = 0.1259; *n* = 7 in each group). (j) Duration in the central zone during the OFT (*t*‐test, *p* = 0.0350; *n* = 7 in each group). (k) Open arm ratio in the EPM (*t*‐test, *p* = 0.0487; *n* = 7 in each group). (l) Sucrose preference ratio in the SPT (*t*‐test, *p* = 0.3093; *n* = 7 in each group). (m) Representative images showing CaMKII^+^ neurons, c‐Fos^+^ neurons, and co‐localized cells in the PrL of each group. Scale bar: 100 μm. (n) Number of c‐Fos^+^ neurons co‐localized with CaMKII^+^ neurons (*t*‐test, *p* = 0.0016; *n* = 3 slices from 3 rats per group).

Conversely, infliximab (INF), a clinically approved TNF monoclonal antibody [35] was infused into the dmPFC of CUMS rats (Figure 6h). INF increased OFT center‐zone duration and the EPM open‐arm ratio compared with the CUMS group, whereas the sucrose preference ratio remained unchanged (Figure 6j–l). It also reduced dmPFC *Tnf* expression and increased the number of c‐Fos/CaMKIIα co‐labeling cells (Figure S5b and Figure 6m,n). Together, these bidirectional pharmacological findings suggest that CWJ attenuates stress‐related behavioral abnormalities by reducing TNF‐related inflammatory signaling and enhancing dmPFC glutamatergic neuronal activity.

## 4 Discussion

CWJ attenuated stress‐related behavioral abnormalities in CUMS rats, which was associated with the restroration of dmPFC neuronal activity and suppression of the TNF‐related signaling pathway. Pharmacological manipulation of TNF‐related inflammatory signaling further supported the contribution of this pathway to the behavioral effects of CWJ. These findings link TNF‐related neuroinflammation to dmPFC glutamatergic dysfunction and suggest that this interaction contributes to the effects of CWJ.

Traditional Chinese Medicine offers various interventions to ameliorate stress‐related behavioral abnormalities. For instance, music interventions and Chinese herbs have been shown to alleviate stress‐induced anxiety‐ and depression‐like behaviors, accompanied by modulation of neuroendocrine, inflammatory, and neurotrophic pathways [16, 36]. Previous pharmacological studies have also shown that ASH and SC possess anti‐inflammatory, antioxidative, anti‐stress, anti‐fatigue, and neuroprotective properties [19, 37, 38, 39, 40]. However, compared with studies on the individual herbs, the pharmacological effects of their combined application remain insufficiently characterized. In the present study, CWJ attenuated CUMS‐induced anxiety‐ and depression‐like behaviors and HPA axis hyperactivity, as reflected by significant changes in the EPM, FST, and serum CORT levels, supporting the therapeutic potential of this herbal combination in stress‐related affective disturbances. Previous studies have shown that bioactive components of ASH enhance the protein expression of brain‐derived neurotrophic factor (BDNF) in the hippocampus and promote anxiolytic behavior [17, 41], whereas schisandrol from SC exerts antidepressant‐like effects through the modulation of the brain‐liver‐gut axis [37]. Collectively, these observations suggest that the antidepressant‐like effects of CWJ may arise from the coordinated actions of multiple bioactive constituents.

Depression is associated with dysfunction across multiple interconnected brain regions, including the prefrontal cortex, lateral habenula, and hippocampus [42, 43, 44]. Among these regions, the dmPFC has been widely implicated in the pathophysiology of MDD, consistent with our previous findings [21, 45]. In the present study, rs‐fMRI revealed functional alterations involving the dmPFC, and c‐Fos immunostaining showed altered neuronal activity in the PrL. Because no additional acute stress stimulus was applied before tissue collection, these findings may reflect the long‐term effects of CUMS exposure and CWJ intervention on the activity‐related neuronal state of the dmPFC. Consistent with this interpretation, fiber photometry demonstrated that CUMS reduced glutamatergic neuronal activity in the dmPFC, and this reduction was attenuated by CWJ treatment. Furthermore, the selective chemogenetic inhibition of dmPFC glutamatergic neurons weakened the antidepressant‐like effects of CWJ, supporting the functional involvement of these neurons in CWJ‐mediated behavioral improvement. Collectively, these findings suggest that a normalization of stress‐induced dmPFC glutamatergic hypoactivity may constitute an important neural substrate underlying CWJ effects. After identifying the dmPFC as a candidate brain region involved in the effects of CWJ, we further explored its molecular basis using transcriptomic analysis and found that TNF‐related inflammatory signaling was among the  pathways imolicated in the response of CWJ. Consistent with the findings of previous studies showing anti‐inflammatory and neuroprotective properties of CWJ constituents [46], our data demonstrated that CWJ significantly reduced TNF‐α expression, attenuated microglial overactivation, and alleviated synaptic impairment in the dmPFC. These finding are of particular interest because growing evidence indicates that inflammatory and immune dysregulation contributes to the pathogenesis of depression.

TNF has emerged as an important inflammatory mediator in the neurobiology of depression [47]. Clinical and preclinical studies have linked TNF dysregulation to several psychiatric disorders, particularly depression, in which elevated TNF‐α levels are associated with greater symptom severity [12, 13, 44]. Notably, TNF antagonism has been reported to improve effort‐based decision‐making in depressed patients with increased inflammatory burden [48], although such approaches may be most relevant to patients with inflammation‐related depressive subtypes rather than depression in general [49]. Therefore, whether TNF signaling represents a potential target for alleviating CUMS‐induced depression‐like behaviors requires further investigation. We addressed this issue by pharmacologically modulating TNF‐related inflammatory signaling in the dmPFC. Inhibition of TNF with INF attenuated CUMS‐induced behavioral abnormalities and increased CaMKIIα/c‐Fos co‐labeling, suggesting that excessive TNF‐related signaling is associated with both stress‐related behavioral changes and reduced activity of dmPFC glutamatergic neurons. Conversely, R848 activates TLR7/8‐NF‐κB innate immune signaling and promotes the induction of pro‐inflammatory cytokines, including TNF‐α [50]. Consistent with this mechanism, local R848 administration significantly increased TNF‐α mRNA expression in the dmPFC, weakened the behavioral effects of CWJ, and reduced the number of CaMKIIα/c‐Fos double‐positive neurons.

Taken together, our findings suggest that CWJ modulates both dmPFC glutamatergic neuronal activity and TNF‐associated neuroinflammatory signaling under chronic stress. However, these results do not show that TNF directly controls glutamatergic neuronal firing or acts as the only upstream mediator. Instead, they suggest that CWJ may preserve dmPFC glutamatergic neuronal function, at least in part, by suppression of TNF‐associated neuroinflammation.

This study has some limitations. First, the lack of significant pairwise differences in the sucrose preference ratio suggests that the anhedonia‐like phenotype should be interpreted cautiously. Second, only male rats were used, and the effects of CWJ in females remain to be determined. Third, although rs‐fMRI was performed under standardized anesthetic and physiological conditions, potential anesthesia‐related effects and the limited sample size should be considered. Fourth, although mCherry controls receiving the same CNO treatment were included in the chemogenetic experiments, potential DREADD‐independent effects of CNO cannot be completely excluded. Fifth, after applying Benjamini–Hochberg correction, only 4 genes met the combined criteria of an adjusted *p* < 0.05 and an absolute |log_2_ fold change| > 0.585 in the CUMS‐versus‐CTRL comparison. Therefore, our current transcriptomics results are only an exploratory analysis. Finally, the bioactive constituents and metabolites responsible for CWJ's effects remain unidentified, warranting future studies integrating pharmacokinetic profiling with compound‐specific validation and TNF/TNFR‐targeted functional approaches.

## 5 Conclusion

In summary, the present study provides evidence that CWJ exerts antidepressant‐like effects in CUMS rats through modulation of dmPFC neuronal activity and TNF‐related neuroinflammation. By partially restoring glutamatergic neuronal function, suppressing the TNF‐related signaling pathway, and reducing microglial overactivation in the dmPFC, CWJ ameliorated stress‐induced behavioral abnormalities. These findings expand current understanding of the neuropharmacological mechanisms of CWJ and support further preclinical investigation of CWJ as a potential intervention for depression.

## Author Contributions

Zhen Wang and Dongdong Shi designed the study, and Fei Tian wrote the article. Fei Tian, Yi Li, Mengyu Dai, Yihan Guo, Yingdan Zhang, Bingbing Liao, and Xin Gong performed the animal experiments. Fei Tian and Yi Li collected and analyzed the data. Zhen Wang, Lanying Liu, and Dongdong Shi coordinated the study. Fei Tian edited and Dongdong Shi revised the manuscript. Zhen Wang and Dongdong Shi proved final version of the manuscript. All authors critically revised the article or contributed important intellectual content.

## Funding

This work was supported by the National Natural Science Foundation of China, 32271066, 82471532. Shanghai Science and Technology Committee, 23QA1408300, [2023]24. National Administration of Traditional Chinese Medicine, ZDYN‐2024‐A‐013. Shanghai Mental Health Center, QNPI002. Shanghai Municipality Administration of Traditional Chinese Medicine, shzyyzdxk‐2024107.

## Ethics Statement

All animal procedures were approved by the Institutional Animal Care and Use Committee of Shanghai Jiao Tong University (approval no. A2024334).

## Conflicts of Interest

The authors declare no conflicts of interest.

## Supporting information


**Table S1:** List of PCR primers.
**Figure S1:** OFT center‐duration analysis adjusted for locomotor activity. The scatter plots show the center duration as a function of total distance. Each point represents a single rat. The lines indicate the additive ANCOVA model fit after testing for group × distance interaction. (a) In the CUMS group, the group × distance interaction was not significant [F(1, 13) = 0.351, p = 0.5635], supporting the homogeneity of the slope assumption. After adjusting for total distance, the group effect on center duration was not significant [F(1, 14) = 0.794, p = 0.3881], and the distance effect was also not significant [F(1, 14) = 1.565, p = 0.2315]. (b) In the CWJ‐treated group, the group × distance interaction was not significant [F(1, 14) = 0.104, p = 0.7516]. After adjusting for total distance, the group effect on center duration remained non‐significant [F(1, 15) = 1.153, p = 0.2999], whereas total distance was significantly associated with center duration [F(1, 15) = 6.814, p = 0.0197].
**Figure S2:** UPLC‐QTOF‐MS‐MS profiles of CWJ. (a) Positive mode. (b) Negative mode.
**Figure S3:** Targets identified through network pharmacological prediction include: (a) a Venn diagram showing intersecting targets of CWJ and depression; (b) a protein–protein interaction (PPI) network of core intervention targets; (c) GO pathway analysis of potential CWJ targets in depression treatment; (d) KEGG pathway enrichment analysis of potential CWJ targets in depression treatment.
**Figure S4:** CWJ attenuated CUMS‐induced changes in inflammatory‐factor expression in the dmPFC. (a) RT‐qPCR analysis of Il4, Ifng, Tnf, Il6, and Il10 expression in the CTRL, CUMS, and CWJ groups. Data are presented as mean ± SEM; *n* = 3 per group. Statistical significance was determined by one‐way ANOVA followed by Dunnett's multiple‐comparisons test, with the CUMS group as the reference group. Compared with the CUMS group, CWJ significantly reduced IFN‐γ (p = 2.35 × 10−5), TNF‐α (p = 0.00069), and IL‐6 (p = 0.00935), and increased IL‐10 (p = 0.00019). The increase in IL‐4 after CWJ treatment did not reach statistical significance (p = 0.097).
**Figure S5:** TNF‐α mRNA expression under TNF‐related pharmacological manipulation. (a) TNF‐α mRNA expression was increased in the R848 group compared with the CWJ group (t‐test, p < 0.0001). (b) TNF‐α mRNA expression was decreased in the INF group compared with the CUMS group (t‐test, p = 0.0008).

## Data Availability

The data that support the findings of this study are available from the corresponding author upon reasonable request.
