# Supplementary material for: Compound Ciwujia Granules Attenuate Stress‐Induced Anxiety‐ and Depression‐Like Behaviors via Inhibition of TNF‐α‐Associated Neuroinflammation in the dmPFC
Source: CNS Neurosci Ther. 2026 Sep 30;32(10):e71178. doi: 10.1002/cns.71178 (PMC13627883; doi:10.1002/cns.71178)
Supplement: Supplementary file 1 [file CNS-32-e71178-s001.zip › cns71178-sup-0001-TableS1-FigureS1@R4-Supplementary materials.docx]

**Supplementary materials**

**Table S1** List of PCR primers

| **Gene** | **Oligonucleotides** |
| --- | --- |
| *Gapdh* | Forward: AAGGTCGGTGTGAACGGATT |
|  | Reverse: TGAACTTGCCGTGGGTAGAG |
| *Il6* | Forward: AGAGTCACAGAAGGAGTGGC |
|  | Reverse: GGTTTGCCGAGTAGACCTCAT |
| *Il4* | Forward: CCTTGCCTGTCACCCTGTTC |
|  | Reverse: CGTTCTCGGTGGTTTCCTT |
| *Il10* | Forward: CAGCAAAGGCCATTCCATCC |
|  | Reverse: GCTTGGCAAACCCAAGTAACC |
| *Tnf* | Forward: ATCCGAGATGTGGAACTGGC |
|  | Reverse: CGATCACCCCGAAGTTCAGT |
| *Ifng* | Forward: GGCAAAAGGACGGTAACACG |
|  | Reverse: TTCACCTCGAACTTGGCGAT |

**Fig. S1 OFT center-duration analysis adjusted for locomotor activity.** The scatter plots show the center duration as a function of total distance. Each point represents a single rat. The lines indicate the additive ANCOVA model fit after testing for group × distance interaction. (**a**) In the CUMS group, the group × distance interaction was not significant (F(1, 13) = 0.351, P = 0.5635), supporting the homogeneity of the slope assumption. After adjusting for total distance, the group effect on center duration was not significant (F(1, 14) = 0.794, P = 0.3881), and the distance effect was also not significant (F(1, 14) = 1.565, P = 0.2315). (**b**) In the CWJ-treated group, the group × distance interaction was not significant (F(1, 14) = 0.104, P = 0.7516). After adjusting for total distance, the group effect on center duration remained non-significant (F(1, 15) = 1.153, P = 0.2999), whereas total distance was significantly associated with center duration (F(1, 15) = 6.814, P = 0.0197)

**S-Figure 2: UPLC-QTOF-MS-MS profiles of CWJ.** (a) Positive mode. (b) Negative mode

**S-Figure 3: Targets identified through network pharmacological prediction include:** (a) a Venn diagram showing intersecting targets of CWJ and depression; (b) a protein-protein interaction (PPI) network of core intervention targets; (c) GO pathway analysis of potential CWJ targets in depression treatment; (d) KEGG pathway enrichment analysis of potential CWJ targets in depression treatment.

**S-Figure 4: CWJ attenuated CUMS-induced changes in inflammatory-factor expression in the dmPFC. (**a) RT-qPCR analysis of Il4, Ifng, Tnf, Il6, and Il10 expression in the CTRL, CUMS, and CWJ groups. Data are presented as mean ± SEM; n = 3 per group. Statistical significance was determined by one-way ANOVA followed by Dunnett’s multiple-comparisons test, with the CUMS group as the reference group. Compared with the CUMS group, CWJ significantly reduced IFN-γ (P = 2.35 × 10⁻⁵), TNF-α (P = 0.00069), and IL-6 (P = 0.00935), and increased IL-10 (P = 0.00019). The increase in IL-4 after CWJ treatment did not reach statistical significance (P = 0.097).

**S-Figure 5. TNF-α mRNA expression under TNF-related pharmacological manipulation.** (a) TNF-α mRNA expression was increased in the R848 group compared with the CWJ group (t test, P ＜ 0.0001). (b) TNF-α mRNA expression was decreased in the INF group compared with the CUMS group (t test, P = 0.0008).
